# Pregnancy Complicated by Maternal MODY 3 and Paternal MODY 2 Diabetes and Subsequent Rapidly Falling Insulin Requirement

**DOI:** 10.1155/2018/9451061

**Published:** 2018-09-26

**Authors:** Anastasia Mikuscheva, Adel Mekhail, Benjamin J. Wheeler

**Affiliations:** ^1^Gynecology and Obstetrics, Southern District Health Board, Dunedin, New Zealand; ^2^Department of Women's and Children's Health, University of Otago, Dunedin, New Zealand; ^3^Paediatric Endocrinology, Southern District Health Board, Dunedin, New Zealand

## Abstract

*Background*. ‘Maturity-Onset Diabetes of the Young' (MODY) or monogenic diabetes accounts for approximately 1–2% of diabetes and is frequently misdiagnosed as type 1 or type 2 diabetes. Here we report a case of a 19-year-old pregnant woman with a MODY 3 diabetes expecting a child to a father with MODY 2 diabetes. Possible inheritance scenarios are described and the implications of these scenarios on the pregnancy and infant are discussed. In addition, the pregnancy was complicated by drastically falling insulin requirements in the mother in the 3^rd^ trimester as well as preterm labour and delivery at 33+4 weeks of gestation.

## 1. Introduction

Monogenic forms of diabetes are thought to be responsible for approximately 2% of all diabetes cases diagnosed before the age of 45 years [1, 2]. Approximately 80% of cases are misdiagnosed as either type 1 or type 2 diabetes, reflecting lack of physician awareness and/or access to genetic testing [3]. Clues to the diagnosis of monogenic forms of diabetes include lack of typical characteristics of type 1 diabetes (no autoantibodies, low or no insulin requirement five years after diagnosis, persistence of stimulated C-peptide of 4200pmol/L, and absence of diabetic ketoacidosis), or type 2 diabetes (lack of obesity, hypertension, and dyslipidaemia), in the presence of a strong family history [1]. There are at least 13 subtypes of Maturity-Onset Diabetes of the Young (MODY) known to date. They are usually characterized by an early onset, autosomal dominant mode of inheritance, and a primary defect in pancreatic *β*-cell function [4], the most common of which are outlined in Table 2. Making a specific diagnosis of MODY can have important implications on patient treatment, prognosis, and genetic counselling. There are also implications for management of pregnancy in affected females. Depending on the MODY subtype different complications may arise and different therapies and monitoring options may apply [2].

Here we present the rare, and previously not described, circumstance of a pregnancy where both unrelated parents were each affected by a different autosomal dominant form of MODY. The outcome and potential clinical implications to both the pregnancy and the child are discussed.

## 2. Case Report

**Figure 1:**
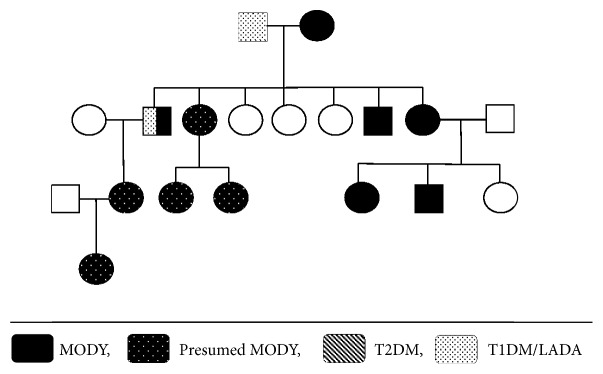
Family tree paternal Glucokinase mutation [7].

Miss S, a 19-year-old woman, presented to antenatal clinic at 19 weeks gestation for a first consultation because of a preexisting hepatocyte nuclear factor *α* (HNF-1*α*) mutation causing MODY 3 diabetes. The patient was well known to the paediatric endocrinology and diabetes services since age of 11 years when her condition first became apparent through recurrent mucosal candidiasis and mild postprandial hyperglycaemia. Due to a strong family history of diabetes (Figure 1) and negative testing for type I diabetes, an HNF1*α* gene mutation was suspected and subsequently confirmed on molecular genetic testing. Interestingly, in addition to a known pathogenic mutation, she also had a second missense variant in HNF1*α* of uncertain clinical significance (Table 1). The patient was initially successfully treated with the sulfonylurea (SU) gliclazide, which more recently was switched to insulin due to increasing hyperglycaemia.

**Figure 2:**
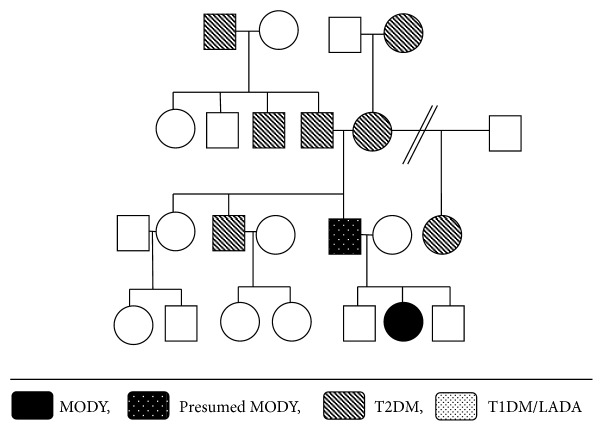
Family tree maternal HNF1-*α* mutation [7].

The father of the fetus is a 21-year-old man also well known to endocrinology and diabetes teams from age of 9 years, due to persistent mild hyperglycaemia and very significant family history of diabetes (Figure 2). Genetic testing for a Glucokinase (GCK) mutation was performed and confirmed the presence of MODY 2 diabetes (Table 1). Following diagnosis, as anticipated, the father remained asymptomatic and did not require any further treatment.

Prenatally, given the autosomal dominant inheritance pattern of MODY, the inheritance possibilities were calculated as follows: 25% chance of being healthy without any form of MODY, 25% chance of having MODY 2 only, 25% chance of having sole MODY 3, and 25 % chance of having compound heterozygous mutations for both MODY 2 and MODY 3. From a pregnancy point of view, a plan was made for biweekly growth scans starting at 24 weeks of gestation and to review the patient fortnightly in combined obstetric and diabetes clinic. Pregnancy targets are individualised in this clinic, but in general aim for fasting glucose <5mmol/L; 2 hour postprandial <6.7mmol/L. She was managed with insulin glargine (Lantus®) daily and insulin aspart (Novorapid®) with meals, and insulin requirements gently increased over the pregnancy, from approximately 0.75 units/kg/day early pregnancy to 0.83 units/kg day at 28 weeks.

Despite this relatively small increase in dosing, her HbA1c fell from a pre-pregnancy value of 68mmol/mol (8.4%) to 45mmol/mol at 18 weeks, and 35mmol/mol at 28 weeks. From 28 weeks, doses were further reduced, until she presented to the emergency department at 33+3 weeks of gestation for frequent hypoglycaemia. She was admitted to the antenatal ward and her insulin was gradually reduced from an approximate total daily insulin dose 0.65 units/kg/day to glargine 4 units and 1 unit of aspart per 18g of carbohydrates with meals (total daily dose approximately 0.28 units/kg/day). Given the significant fall in insulin requirement, concerns were raised that this might be due to a failing fetoplacental unit. Against this, the patient never developed hypertension, and laboratory screening for preeclampsia was performed multiple times and was always within normal limits.

Fetal growth until this presentation had been measured by fortnightly ultrasound and was on the 50^th^ centile of the Australasian Society of Ultrasound in Medicine (ASUM) growth charts. Given the unknown significance of the falling insulin requirements, biweekly monitoring of fetal wellbeing via Doppler measurements was commenced, which was satisfactory at all times. The patient received 2 doses of intramuscular Betamethasone 11.4 mg intramuscularly for lung maturation. At 34+3 weeks of gestation the patient went into spontaneous labour and delivered a healthy baby girl via forceps, weight 2.22kg, APGARs 7, 9, and 10 (at 1, 5, and 10 min, respectively). Histological examination of the placenta was not performed. Due to prematurity, the baby was admitted to the neonatal intensive care unit and was discharged home at 36+1 weeks of life. Postnatal genetic testing in the baby showed a heterozygous mutation for the maternal familial likely nonpathogenic HNF1A gene variant (Table 1), which has been reported in the literature with two functional studies and found to be of uncertain clinical significance [5, 6]. Importantly, both parental known pathogenic mutations were absent.

## 3. Discussion

This case presents the not previously reported chance possibility of a child inheriting compound heterozygous monogenic diabetes mutations from unrelated parents affected by two different forms of MODY. This is informative and illustrates a number of possible outcomes. Given the fact that both parents are carriers of heterozygous mutations, the chances of the fetus inheriting the maternal pathogenic HNF1*α* mutation were 25%. In most cases a mother affected by MODY 3 can safely be treated with low dose sulfonylurea throughout pregnancy as outlined above; the mechanism of action has been described elsewhere [2]. Unfortunately, in our case the patient did not achieve sufficient glycaemic control with sulfonylureas. It remains unclear why this is the case; one possibility could be the influence of the mother's 2^nd^ mutation (Table 1), considered nonpathological. In the case where mother and fetus are affected by MODY 3 there would not be any additional implications for the fetus during pregnancy other than those associated with diabetes in general. Unlike HNF4A (MODY 1), mutations, HNF1A mutations are not associated with an increased birthweight [8].

The chance of the fetus inheriting the paternal GCK mutation was equally 25%. Patients with a defect in one copy of their GCK gene (MODY 2) have fasting hyperglycaemia that may be present from birth and show very little deterioration with age [9]. The diagnosis is often made incidentally, for instance, during routine pregnancy gestational diabetes screening [2]. In a pregnancy where the mother is affected by MODY 2 the fetus will not inherit the GCK mutation in 50% of cases, and will respond to maternal hyperglycaemia by excess insulin production and therefore excess growth (by approximately 550–700g) [2]. Alternatively, if the fetus does inherit the GCK abnormality it will sense the maternal hyperglycaemia as normal, produce normal amounts of insulin, and have normal growth [2]. A case of a mother with MODY 3 carrying a fetus with MODY 2 has not been described in the literature and hence the implications of this are unknown. However, extrapolating from the scenario where mother and fetus are both affected by MODY 2, a fetus with MODY 2 to a mother with MODY 3 would be able to tolerate maternal hyperglycaemia better than a fetus with no MODY.

The chances of the fetus inheriting a MODY 2 and MODY 3 compound mutation were equally 25%. This constellation has not previously been described in the literature and hence the implications of it in pregnancy and to the fetus are not well described, but would likely result in classic MODY 3 eventually with the additional complication of an altered glucose setpoint—this would need to be taken into account when setting realistic glycaemic targets. More usually, or rarely, the clinically more serious situation of inheriting homozygous mutations from a consanguineous union occurs. This later situation has been previously reported for a homozygous GCK mutation leading to permanent neonatal diabetes [10].

The unknown aetiology and relevance of the dramatically falling insulin requirements (FIR) in our patient from 0.83 – 0.28 units/kg/day before 34 weeks of gestation caused considerable concern amongst the team of obstetricians and endocrinologists looking after her. The question whether FIR represent a marker of placental insufficiency and should hence lead to obstetric intervention such as induction of labour has repeatedly been addressed in the non-MODY literature. One study found that FIR of ≥15% increased the risk of preeclampsia by more than 6-fold and the babies of affected women were more likely to be delivered early by emergency caesarean section and admitted to the NICU. However, there was no difference in the levels of hormones mediating insulin resistance [11, 12] which is in keeping with most studies published to date. This does not seem to support the historical theory of FIR being a sign of placental insufficiency. Most studies on the subject did not find FIR to cause any adverse neonatal outcome [13–16]. However, all of these studies were retrospective and apart from [15] comprised small patient numbers. Based on the above findings the current clinical recommendation for all women manifesting FIR ≥15% is for increased surveillance and investigation for adverse obstetric outcomes; however, its presence does not necessarily indicate urgent, immediate delivery [11]. This was the only prospective multicentre study including 158 women and hence adequately powered. A case of a large decrease in insulin requirements (over 50%) in the final few weeks of two gestations in a woman with type 1 diabetes mellitus has been reported. The first pregnancy was otherwise uneventful and delivered spontaneously at 39 weeks; the 2^nd^ pregnancy was complicated by preeclampsia which required induction of labour at 37 weeks [17]. However, none of the above literature relates to MODY affected pregnancies. In this case, as the first report, it remains uncertain if the falling insulin requirements were a product of the pregnancy itself, and possible underlying placental dysfunction, or whether there was an additional HNF1-alpha affect, via placental hormones/pregnancy directly impacting beta cell function.

## 4. Conclusion

The optimal care for pregnant patients with MODY mutations is multidisciplinary and should involve obstetricians, endocrinologists, geneticists, and paediatricians. In a constellation where both parents are affected by different MODY mutations, with noninvasive prenatal testing covering more and more genetic conditions, it will hopefully be possible to determine prenatally if and to what extent the fetus is affected by the parents' mutation and thus provide optimal counselling and pregnancy care. The significance of falling insulin requirements in pregnancy is a debated subject in the literature and its significance is not entirely clear to date. Moreover, due to the small prevalence of MODY mutations in the population, it is not clear whether the available data for GDM and types I and II diabetes can easily be extrapolated to MODY patients.

## Figures and Tables

**Table 1:** 

Paternal mutation	One copy of the variant c.698G>A (p.Cys233Tyr) in exon 7 of the GCK gene (Refseq accession number NM_000162)

Maternal mutation	Frameshift mutation c.864delGinsCC, or c.864G>C and c.872dupC, (p.Gly292ArgfsX25) in exon 4 of the HNF1*α* gene (Refseq accession number NM_000545)

Fetal mutation	c.[92G>A] (p.[(Gly31Asp)]1

**Table 2:** MODY subtypes and pregnancy implications (4 most common subtypes in descending order of frequency).

**Gene and MODY subtype in the mother**	***Gene Function *+ Phenotype**	**Prognosis**	**Associated pregnancy implication**
HNF1-alpha gene (MODY 3)	*Regulates insulin gene transcription* Reduced insulin secretion/diabetes and marked sensitivity to sulfonylurea	Progressive May require insulin May develop secondary complications	Not associated with increased birthweight
Glucokinase (GCK) gene (MODY 2)	*Catalyses conversion of glucose to glucose-6-phosphate* Reduced glucose sensing by beta cells – Mild diabetes	Generally non or slowly progressive Complications rare	Unaffected fetus—Excess fetal growth if no GCK mutation Affected fetus—maternal hyperglycemia will be sensed as normal and result in normal growth
HNF4-alpha gene (MODY 1)	*Nuclear transcription factor that regulates hepatic and pancreatic beta cell gene expression * Reduced insulin secretion/diabetes and marked sensitivity to sulfonylurea	Progressive May require insulin May develop secondary complications	Associated with increased birth weight (50% of babies), can cause neonatal hyperinsulinaemic hypoglycaemia
HNF1-beta gene (MODY 5)	*Regulates HNF4á gene transcription* Insulin resistance + wide clinical spectrum +/- Urogenital/pancreatic anomalies +/- Pancreatic exocrine failure +/- Developmental delay/Learning difficulties	Progressive beta-cell failure with diabetes onset around puberty Insulin resistance without obesity Insulin dependence	In affected mother—possible pregnancy complications associated with genital and uterine malformations, such as recurrent miscarriages or preterm labour For affected fetus—Urogenital malformations may be visible on prenatal ultrasound

Table 2 adapted from [7].

## Data Availability

No data were used to support this study.
